# Effectiveness of a Personalized Digital Exercise and Nutrition Rehabilitation Program in Postoperative Patients With Gastric Cancer: Randomized Controlled Trial

**DOI:** 10.2196/85795

**Published:** 2026-02-26

**Authors:** Inah Kim, Ji Young Lim, Sun Woo Kim, Jun Ho Lee, Tae Sung Sohn, Sungsoo Park, Seok Ho Kang, Ji Youl Lee, Ji Hye Hwang

**Affiliations:** 1 Department of Rehabilitation Medicine Dongtan Sacred Heart Hospital Hallym University College of Medicine Hwaseong-Si Republic of Korea; 2 Department of Physical and Rehabilitation Medicine School of Medicine Sungkyunkwan University Seoul Republic of Korea; 3 Department of Surgery Samsung Medical Center Sungkyunkwan University School of Medicine Seoul Republic of Korea; 4 Department of Surgery Korea University Anam Hospital Korea University College of Medicine Seoul Republic of Korea; 5 Department of Urology Korea University Anam Hospital Korea University College of Medicine Seoul Republic of Korea; 6 Department of Urology Seoul St. Mary's Hospital College of Medicine, The Catholic University of Korea Seoul Republic of Korea; 7 Department of Physical and Rehabilitation Medicine Samsung Medical Center Sungkyunkwan University School of Medicine Seoul Republic of Korea

**Keywords:** digital health, eHealth, gastric cancer, mHealth, randomized controlled trial, rehabilitation

## Abstract

**Background:**

Nutritional and exercise interventions have shown beneficial effects after gastrectomy for gastric cancer. While digital health tools show promise in cancer care, their long-term effectiveness in patients with gastric cancer remains unclear. In addition, large-scale studies of personalized interventions initiated in the immediate postoperative period are lacking.

**Objective:**

This study aimed to determine whether a personalized mobile health intervention incorporating exercise and nutrition confers additional benefits in weight change, body composition, nutritional status, physical fitness, and quality of life compared with standard care when applied continuously for 12 months after gastrectomy.

**Methods:**

This multicenter, randomized controlled trial enrolled 257 patients who had undergone curative resection for stage I-III gastric cancer. Participants were randomly assigned (2:1) to either a 12-month personalized mobile health intervention group or a standard rehabilitation control group. The digital intervention incorporated a smartphone app and wearable device, offering exercise and dietary plans tailored to clinical parameters such as BMI, surgery type, and recovery stage. Standard care, including nutritional education, was provided to all patients. The primary outcome was a change in body weight over 12 months. Secondary outcomes included quality of life (European Organisation for Research and Treatment of Cancer Quality of Life Questionnaire-Core 30 [EORTC QLQ-C30] and European Organisation for Research and Treatment of Cancer Quality of Life Questionnaire-Stomach 22), nutritional status (Mini Nutritional Assessment Score), physical fitness (grip strength, 30-second chair stand test, and 2-minute walk test), physical activity (International Physical Activity Questionnaire-Short Form), pain intensity (average Numeric Rating Scale), body composition (skeletal muscle mass, lean body mass, and fat mass), BMI, hemoglobin, vitamin B_12_, and albumin. Assessments were conducted at baseline and at 1, 3, 6, and 12 months postoperatively.

**Results:**

No significant group-by-time effects were observed for weight change. Secondary outcomes showed no between-group differences, except for 1 subscale of the EORTC QLQ-C30, which lacked clinical significance. The intervention group reported high satisfaction and adherence to the mobile app, and no adverse events or incidents were observed during the 12-month study period.

**Conclusions:**

The digital health program integrating exercise and nutrition was safe and feasible, with high satisfaction and adherence among patients with gastric cancer. However, it was not superior to standard education in modifying the postoperative trajectory in patients with gastric cancer after surgery, including body weight change and related functional or nutritional outcomes. These findings suggest that future digital health programs should be precisely targeted and tailored to specific patient populations and recovery phases.

**Trial Registration:**

ClinicalTrials.gov NCT04907591; https://clinicaltrials.gov/ct2/show/NCT04907591

## Introduction

In patients with gastric cancer (GC) undergoing gastrectomy, prevention of postoperative weight loss is a critical factor for reducing complications and improving both cancer-specific survival and quality of life [1,2]. However, malnutrition is highly prevalent, affecting more than half of patients who are affected or at risk [3]. Nutritional impairment leads to weight loss and skeletal muscle depletion, which in turn are associated with reduced activities of daily living and poorer survival outcomes [4,5]. Evidence indicates that nutritional or exercise interventions in the postoperative course of GC provide beneficial effects [6,7]. For example, randomized controlled trials have demonstrated that dietary interventions significantly attenuate weight loss, particularly in patients undergoing total gastrectomy, with benefits sustained not only at 6-8 weeks but also up to 1 year after surgery [8-10]. Likewise, exercise interventions have been shown to enhance muscular strength, cardiopulmonary function, quality of life, and emotional functioning in this population [11,12].

With recent developments in communication technology, mobile health (mHealth) apps have gained attention as supportive tools in cancer management [13-15]. However, studies of digital health interventions in upper gastrointestinal cancers have shown inconclusive efficacy [16-19]. Most previous studies on digital health with wearable devices focused on limited interventions, such as health programs applied only during chemotherapy or those focusing mainly on psychological or nutrition-related issues, often with insufficient personalization, short follow-up periods, or a small number of patients [20,21]. Offering uniform content to this population might be inefficient because patient status varies according to diverse factors such as age, surgical method, or therapeutic phase [10,22,23]. In addition, a small number of patients is not sufficiently generalizable to the broader population.

This study aimed to evaluate whether a long-term, personalized digital health intervention initiated after gastrectomy in patients with GC confers superior effects compared with conventional education in modifying the biological postoperative course.

## Methods

### Patients

Participants were recruited between May 17, 2021, and September 19, 2022. In total, 257 patients diagnosed with GC who underwent curative surgery were enrolled in this study. The inclusion criteria were as follows: (1) age of 19-75 years, (2) curative resection for stage I-III GC according to the American Joint Committee on Cancer staging system, (3) ownership of an Android- or iOS-based smartphone, (4) ability to use a mobile app and to attend regular outpatient follow-up visits, and (5) voluntary agreement to participate in the study. Exclusion criteria included the inability to engage in exercise or diet management due to severe comorbidities, neuromusculoskeletal disorders, cognitive or visual impairment, or communication difficulties.

Eligible patients were identified through consultations with the Departments of Surgery and Rehabilitation Medicine. Following surgery, all eligible participants were informed of the study procedures, and written informed consent was obtained from those who agreed to participate voluntarily.

### Trial Design

This prospective, randomized, controlled, multicenter, open-label, 2-arm trial adhered to the SPIRIT (Standard Protocol Items: Recommendations for Interventional Trials) and CONSORT (Consolidated Standards of Reporting Trials) standards (checklist provided in Multimedia Appendix 1). Participants were recruited from 2 university hospitals in South Korea (Samsung Medical Center and Korea University Anam Hospital) and were randomly assigned (2:1 ratio) to either the digital therapeutic or control group using blocked randomization with randomly selected block sizes of 3 and 6. Patients were randomly allocated to 2 groups for 12 months of the rehabilitation program, starting immediately after the operation: a personalized digital therapeutic (intervention) group and a conventional education-based rehabilitation (control) group. Both groups received standard care and conventional dietary education on the prevention of dumping syndrome and the maintenance of muscle mass and weight.

### Personalization Algorithm and Dynamic Adjustment

Participants in the intervention group (personalized digital therapeutic group) were provided with the “Gastric Cancer by a Second Doctor” mobile app (Medi Plus Solution) and the “DoFit” smart wristband (NF-B20; Medi Plus Solution).

Participants in the usual care group received regular diet management and dumping syndrome prevention training at the hospital.

#### Exercise Prescription Algorithm

The exercise component was individualized using a rule-based algorithm that incorporated patient-specific variables, including weeks since surgery and current chemotherapy status. Based on these factors, participants were categorized into an exercise stage, which determined the target intensity and duration of aerobic activity. The target heart rate (THR) was calculated using the standard formula: THR = (220 – age) × THR factor. The THR factor was defined according to cancer-specific exercise guidelines, including those from the American College of Sports Medicine (2022) and the Roundtable on Exercise for Cancer Survivors (2019), as well as previous studies involving patients with GC, such as the Postoperative Recovery Exercise Program for Gastric Cancer Patients (2018) [12,24,25]. Exercise duration was adjusted according to the assigned stage, and progression was guided by the individual’s clinical recovery. The prescribed exercise program included stretching, flexibility exercises, aerobic training, and functional resistance exercises using body weight or simple equipment. These prescriptions were reviewed and adjusted biweekly based on monitored adherence, patient-reported symptoms, and physiological parameters such as heart rate and step count, allowing real-time tailoring of exercise difficulty. During chemotherapy, low-intensity exercises focused on flexibility were implemented. Additionally, the exercise stage was adjusted upward or downward based on the patient’s self-reported rating of perceived exertion. Table S1 in Multimedia Appendix 2 presents the stepwise logic used for exercise stage progression and adjustment.

#### Nutrition Prescription Algorithm

Nutritional planning was driven by a clinical decision algorithm that considered the patient’s age (<65 vs ≥65 years), extent of gastrectomy (total vs subtotal), postoperative week, and the presence of chronic diseases such as diabetes, hypertension, and dyslipidemia. Caloric requirements were calculated using the formula: ideal body weight × activity factor (26-35 kcal/kg/day). During the early recovery phase (the first 12 weeks postoperatively), calorie targets were conservatively adjusted to reduce the risk of gastrointestinal complications, such as dumping syndrome and diarrhea. After week 13, caloric targets were increased to meet 100% of estimated needs, with additional modifications based on relevant clinical guidelines—such as sodium restriction per the 2022 Korean Society of Hypertension guidelines and carbohydrate adjustment in accordance with the 2021 Korean Diabetes Association recommendations [26,27]. Protein intake was consistently recommended at 1.2 g/kg ideal body weight per day throughout the recovery and adaptation periods, while fat intake was limited to 20% of total energy during early recovery. Meal plans were adapted in real time to reflect changing caloric needs. Specifically, portion sizes and snack frequencies were automatically recalculated. For patients who underwent subtotal gastrectomy, snack frequency was adjusted to accommodate more rapid adaptation to oral intake. Nutritional care followed a phased structure. In the recovery phase (weeks 1-4), energy intake was slightly below estimated needs and focused on soft textures and small, frequent meals. In the adaptation phase (weeks 5-12), patients gradually advanced toward full caloric intake with a transition to regular textures. The maintenance phase (week 13 and beyond) included nutritional modifications based on comorbidities, with targeted adjustments for fat, carbohydrate, sodium, and cholesterol. The corresponding decision rules for energy and nutrient prescription, stratified by surgical type, recovery phase, and clinical profile, are presented in Table S2 in Multimedia Appendix 2.

#### Clinician Monitoring and Feedback

**Figure 1:**
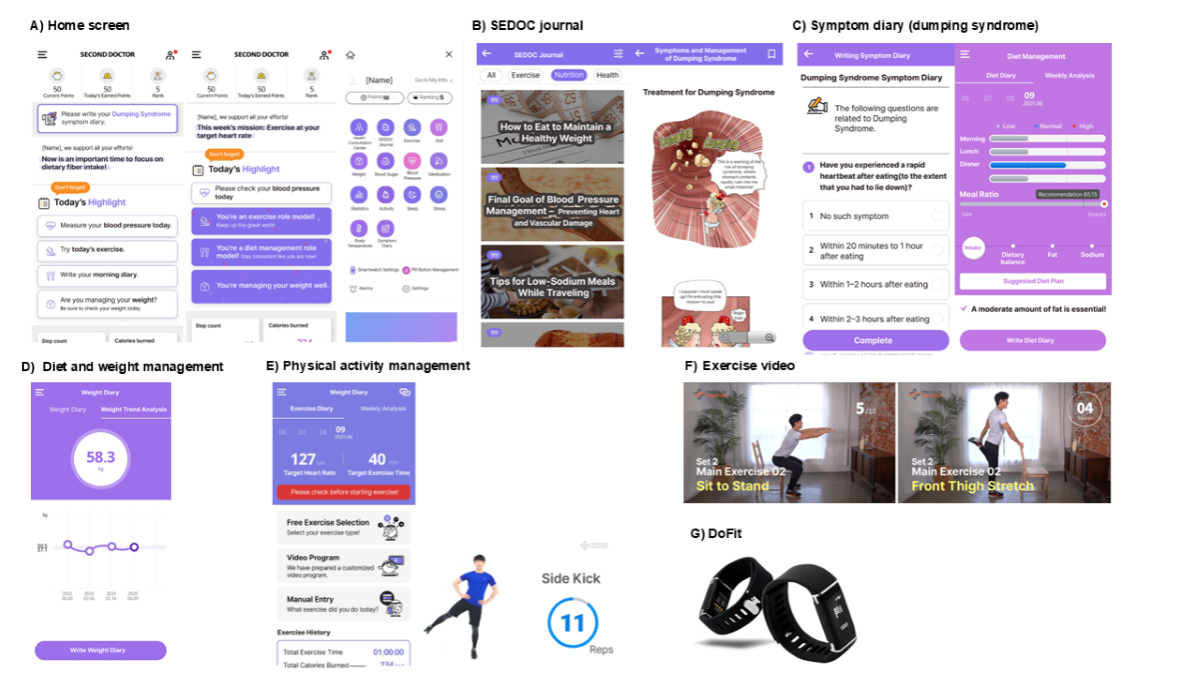
Representative screenshots of the app and smart band interface include (A) main screen displaying today’s to-do list and self-reported symptom monitoring, (B) provision of the general information (Second Doctor [SEDOC] Journal), (C) symptom diary, (D) dietary tracking module and body weight management interface, (E) physical activity tracking module, (F) instructional exercise videos, and (G) DoFit.

The program was designed to operate primarily through a rule-based algorithm, enabling fully automated adjustment of exercise and nutrition prescriptions without requiring direct input from clinicians. A secure, web-based monitoring platform was available to clinical staff (eg, nurses, dietitians, and physiotherapists), allowing access to real-time life-log data, including step count, heart rate, nutritional intake, and symptom reports. While the platform did not allow clinicians to override or alter the algorithmic recommendations manually, it facilitated patient-centered feedback. Clinicians used the data during routine outpatient visits to encourage adherence and self-management, particularly when patients exhibited low engagement or reported challenges. Based on clinical judgment, face-to-face feedback was selectively offered to patients undergoing chemotherapy approximately 3 months postoperatively—when adherence risk is typically higher—to reinforce self-care and address barriers. Screenshots of the representative functions are shown in Figure 1, and the main app functions are listed in Table S3 in Multimedia Appendix 2.

### Outcomes

A trained clinical research coordinator conducted in-person evaluations of the outcome measures during hospital visits. An overview of baseline screening, assessment procedures, and the follow-up schedule across study visits is presented in Table S4 in Multimedia Appendix 2.

#### Primary Outcome

The primary outcome was the change in body weight from baseline to 12 months, assessed using a bioimpedance analysis device to compare the intervention and control groups.

#### Secondary Outcomes

The secondary outcomes included quality of life, cancer-specific symptoms and function, nutritional status, physical activity, physical status, pain intensity, and physical fitness over time.

##### Quality of Life

Health-related quality of life was assessed using the European Organisation for Research and Treatment of Cancer Quality of Life Questionnaire-Core 30 (EORTC QLQ-C30) and European Organisation for Research and Treatment of Cancer Quality of Life Questionnaire-Stomach 22 (EORTC QLQ-STO22). The EORTC QLQ-C30 consists of 30 items, covering functional, symptom, and global quality of life domains. A high EORTC QLQ-C30 score indicates a good quality of life. The EORTC QLQ-STO22, a GC-specific questionnaire, consists of 22 items across symptom domains related to GC [23,28].

##### Nutrition, Pain Intensity, Physical Activity, and Function

###### Korean Mini Nutritional Assessment

Nutritional status was assessed using the Korean version of the Mini Nutritional Assessment (MNA) long form, an internationally validated tool in patients with cancer [29]. Previous studies have shown prognostic value for treatment efficacy, health-related quality of life, mortality, survival, and cancer progression [30]. A total score of less than 17 points indicates malnutrition, 17-23.5 points indicates a risk of malnutrition, and 24-30 points represents the normal range [31].

###### Numeric Rating Scale

Pain intensity over the past week was assessed using an 11-point Numeric Rating Scale (NRS), where 0 indicated “no pain,” and 10 represented “the worst possible pain.” The participants were asked to report both their average and worst perceived pain intensities during this period [32].

###### International Physical Activity Questionnaire-Short Form

Self-reported physical activity was assessed using the International Physical Activity Questionnaire-Short Form (IPAQ-SF) [33]. The IPAQ-SF is a widely used self-report instrument that enables standardized measurement and comparison of physical activity levels across populations. It consists of 7 items assessing the frequency and duration of physical activities performed over the preceding 7 days, including vigorous and moderate activities, walking, and sedentary time [34]. Responses are converted into metabolic equivalent of task (MET) minutes per week, using standardized MET values (8.0 for vigorous activity, 4.0 for moderate activity, and 3.3 for walking) [34,35]. Based on total activity levels, respondents are classified into low, moderate, or high activity categories. Only activities lasting at least 10 continuous minutes are counted [35].

In this study, we used the Korean version of the IPAQ-SF, developed and validated by Oh et al [34], which demonstrated acceptable reliability and validity in Korean adults, including older populations.

###### Grip Strength Test, 30-second Chair Stand Test, and 2-minute Walk Test

Physical fitness was assessed using the grip strength test [36], 30-second chair stand test [37,38], and 2-minute walk test [39], which evaluates the strength generated by the forearm muscles, strength and endurance of the lower extremities, and cardiorespiratory endurance, respectively. Grip strength of the dominant hand was assessed using a handheld dynamometer (microFET Digital HandGRIP Dynamometer; Hoggan Scientific LLC). All tests were performed using standardized measurement methods.

### eHealth Literacy Scale

The eHealth literacy of the intervention group was only measured at baseline using the eHealth Literacy Scale (eHEALS), which comprised 10 items. A total of 8 questions assessed eHealth literacy on a 5-point Likert scale, with higher scores indicating greater literacy [40]. In addition, the intervention group completed a self-developed questionnaire assessing satisfaction with the mobile app and smart band at 6 months.

### App-Based Adherence Measures

“Exercise log (days)” was defined as the number of days in a month on which participants recorded any exercise session—whether free-form, video-guided, or manually entered—that lasted at least 5 minutes. “Exercise duration per exercise day (min)” was calculated by dividing the total exercise time in a month by the number of exercise log days. For trend analyses, participants who had no exercise records in each month were not included in the calculation of mean values. “Step count logged (days)” refers to the total number of days on which step counts were recorded. The “step count” was calculated by dividing the total number of steps over a month by the number of step-log days. To ensure monthly data reflected typical activity patterns, step counts were counted only if data were recorded on at least 7 days within the month. For the trend analysis of step counts, zero values were excluded when calculating the average step count among users with available data. “Weight log” and “diet log” indicate the number of days with at least 1 entry, and “app access” denotes the number of days the user accessed the app at least once.

### Satisfaction Survey

A customized satisfaction questionnaire regarding the use of mobile apps and Internet of Things devices was developed collaboratively by investigators from the 2 participating institutions. This survey was administered exclusively to participants in the intervention group at the 6-month follow-up and used a 5-point Likert scale, with higher scores reflecting greater levels of satisfaction.

### Sample Size Calculation

Using G*Power (version 3.1.9.2; Heinrich Heine University Düsseldorf), the required sample size was calculated for the primary outcome: change in body weight from baseline to 12 months. The primary hypothesis was to test the superiority of the personalized digital intervention in attenuating postoperative percentage body-weight loss. We assumed a standardized effect size (Cohen *d*) of 0.35 (moderate), a significance level (α) of 5%, and power of 80%, based on the characteristics of the intervention described in a previous behavioral intervention study [41]. This effect size was chosen to represent a conservative estimate for the minimum clinically meaningful difference expected from a nonpharmacological, behavioral intervention in a population of cancer survivors. The attrition rate in mobile-based interventions is known to vary depending on the study design. Considering an anticipated dropout rate of 10%, the final target enrollment was set at 324 participants, with 216 assigned to the intervention group and 108 to the control group. In our previous 12-week validation study involving 203 patients with advanced gastrointestinal cancer, approximately 6% discontinued the study for reasons unrelated to changes in physical condition [13].

### Data Handling and Security

Electronic data were captured using a secure web-based platform provided by the Medicallogic Company [42] for systematic and reliable data management. Access to the system was restricted to authorized study personnel.

Participant data were recorded using an electronic case report form, and relevant information from the source documents was entered into the electronic case report form by designated research staff.

### Statistical Analysis

All statistical analyses were conducted using the R software (version 4.4.2; R Foundation for Statistical Computing). Statistical significance was defined as a 2-sided *P* value of <.05, and 95% CIs were calculated. The normality of continuous variables was assessed by examining histograms. For baseline comparisons between the intervention and control groups, the Student *t* test or Mann-Whitney *U* test was used for continuous variables, and the chi-square or Fisher exact tests were applied for categorical variables, as appropriate. For the primary outcome (weight), comparisons at each time point were performed using independent *t* tests, and changes over time were analyzed using mixed-effects models, with group, time, and their interaction included as fixed effects, and participant ID included as a random effect. For secondary outcomes, if the data were normally distributed, mixed-effects models with the same structure (group, time, and group × time interaction as fixed effects, and participant ID as a random effect) were used. For nonnormally distributed outcome measures, the change from 1 to 12 months was compared using the Wilcoxon rank-sum test. To assess the robustness of these findings, supplementary analyses were conducted using generalized estimating equations (GEE), which account for within-participant correlation and allow for nonnormally distributed repeated measures.

Histogram analyses indicated that the following variables were normally distributed: weight, skeletal muscle mass, BMI, skeletal muscle index, total fat mass, grip strength, 30-second chair stand test, and 2-minute walk test. These variables were analyzed using mixed-effects linear regression models, incorporating all available data points at 1, 3, 6, and 12 months, to estimate the slope trajectories.

Conversely, the following variables were nonnormally distributed and were analyzed using the Wilcoxon rank-sum test: NRS average, NRS maximum, vigorous activity, moderate activity, walking time, total METs, MNA subcomponents, and EORTC QLQ-C30 and QLQ-STO22 scores—including physical functioning, role functioning, cognitive functioning, emotional functioning, social functioning, fatigue, nausea/vomiting, pain, dyspnea, insomnia, appetite loss, constipation, diarrhea, financial difficulties, global health status/quality of life, and summary score, as well as dysphagia, reflux, eating restrictions, anxiety, dry mouth, taste alteration, body image, and hair loss. Inflammatory and nutritional biomarkers, such as hemoglobin, vitamin B_12_, and albumin, also showed nonnormal distributions and were analyzed accordingly.

### Safety Monitoring and Discontinuation Criteria

The risk level associated with this study was assessed as minimal based on evaluations conducted by the institutional review board and principal investigator. Throughout the study period, participants were allowed to contact the research team at any time to address study-related concerns. In cases of severe pain or injury, participants underwent a clinical evaluation by the principal investigator. Participants were permitted to withdraw from the study at any time they wished. Additionally, withdrawal could also be initiated by the study team in cases of serious medical conditions or noncompliance with medical instructions.

### Ethical Considerations

All study procedures were reviewed and approved by the institutional review boards of the 2 hospitals (approval numbers SMC-2021-01-090 and 2021AN0104). The trial was registered at ClinicalTrials.gov (approval ID NCT04907591). The original study protocol was submitted to the Samsung Medical Center Institutional Review Board on February 1, 2021, and received approval on February 23, 2021. All collected data were retained for 3 years after study completion and were subsequently destroyed in accordance with institutional policy.

Written informed consent was obtained from all participants prior to their participation, and all participants voluntarily agreed to take part in the study. All data were anonymized and securely stored in restricted-access locations to protect personal information and ensure confidentiality. Participants received a compensation of 50,000 KRW (approximately US $34) after hospital discharge at each follow-up assessment.

## Results

### Patients

A total of 254 patients were included in the intention-to-treat population (131 in the intervention group and 123 in the control group). Overall, 3 participants were excluded from the final analysis due to missing baseline body weight data.

**Figure 2:**
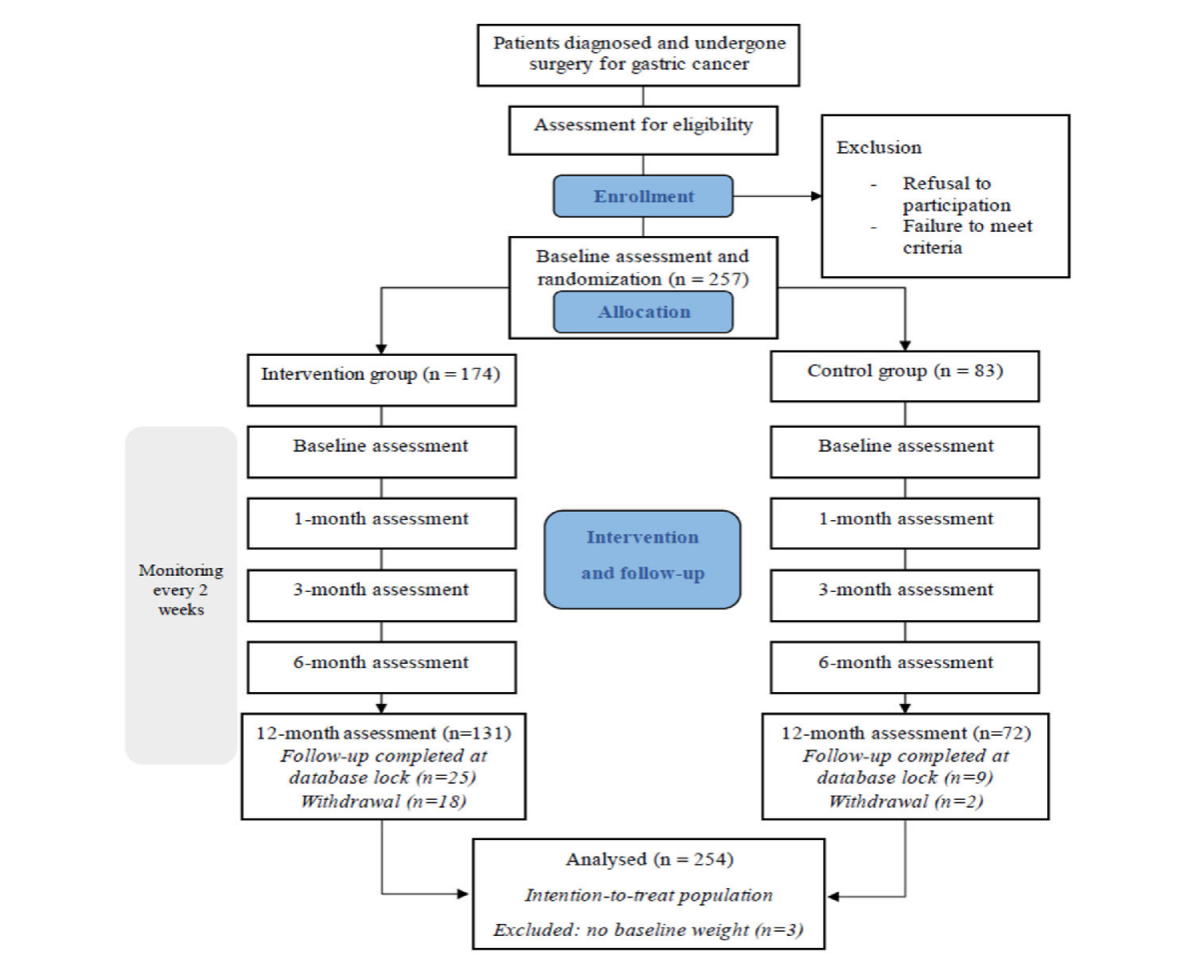
CONSORT (Consolidated Standards of Reporting Trials) flow diagram.

By the 12-month follow-up, several postrandomization dropouts had occurred. In the intervention group, 1 participant died due to lung and brain metastases, and another was diagnosed with non-Hodgkin lymphoma, unrelated to GC. In the control group, dropouts were primarily due to clinical deterioration and failure to attend scheduled follow-up visits. Additional reasons for early withdrawal across both groups included adverse effects from ongoing treatment, allergic reactions to the smartwatch device, and user-reported difficulties with or reluctance to use digital tools. Despite these attritions, all participants were included in the intention-to-treat analysis (Figure 2).

The baseline demographics and clinical variables of the intervention and control groups are shown in Table 1. Baseline characteristics of the participants were well balanced between the 2 groups, except for age, alcohol consumption, adjuvant chemotherapy, and grip strength. To account for baseline imbalances, demographic variables showing between-group differences were included as covariates in the outcome analyses.

**Table 1:** Baseline characteristics of the study participants. Group comparisons were conducted using Student *t* test or Mann-Whitney U test for continuous variables, and the chi-square or Fisher exact tests for categorical variables, as appropriate.

Characteristics			mHealth^a^ (n=174)		Control (n=83)		*P* value	
**Sex, n (%)**								.36
	Female	77 (44.25)		31 (37.35)				
	Male	97 (55.75)		52 (62.65)				
Age (years), mean (SD)			54.91 (9.36)		57.93 (9.15)		.02^b^	
Height (cm), mean (SD)			164.24 (7.88)		164.67 (8.95)		.71	
Weight (kg), mean (SD)			65.35 (11.96)		65.54 (10.60)		.90	
**Smoking, n (%)**								.49
	Current smoker	22 (12.64)		9 (10.84)				
	Ex-smoker	52 (29.89)		31 (37.35)				
	Never smoker	100 (57.47)		43 (51.81)				
**Drinking, n (%)**								.03^b^
	Yes	37 (21.26)		29 (34.94)				
	No	137 (78.74)		54 (65.06)				
**Operation type, n (%)**								.95
	Total gastrectomy	32 (18.5)		14 (17.3)				
	Subtotal gastrectomy	141 (81.5)		67 (82.7)				
**Operation approach, n (%)**								.91
	Laparoscopic	140 (80.46)		68 (81.93)				
	Open	34 (19.54)		15 (18.07)				
**Operation anastomosis, n (%)**								.47
	Billroth 1	31 (17.82)		18 (21.69)				
	Billroth 2	105 (60.34)		44 (53.01)				
	Roux-en-Y	36 (20.69)		18 (21.69)				
	Esophagogastrostomy	1 (0.57)		2 (2.41)				
	Double tract reconstruction	1 (0.57)		1 (1.20)				
**Adjuvant chemotherapy, n (%)**								.03^b^
	No	133 (76.44)		74 (89.16)				
	Yes	41 (23.56)		9 (10.84)				
**Adjuvant radiotherapy, n (%)**								.54
	No	173 (99.43)		82 (98.80)				
	Yes	1 (0.57)		1 (1.20)				
	Yes	11 (6.32)		7 (8.43)				
**Cancer stage, n (%)**								.10
	Stage 1	126 (72.41)		70 (84.34)				
	Stage 2	18 (10.34)		6 (7.23)				
	Stage 3	30 (17.24)		7 (8.43)				
Hb^c^, mean (SD)			13.16 (1.92)		13.53 (1.93)		.16	
Vitamin B_12_, mean (SD)			703.98 (314.85)		670.86 (192.17)		.59	
Skeletal muscle mass, mean (SD)			26.53 (5.79)		27.37 (6.08)		.31	
Skeletal muscle index, mean (SD)			7.09 (1.21)		7.30 (1.16)		.24	
BMI (kg/m^2^), mean (SD)			24.05 (3.36)		24.05 (2.79)		.99	
Total fat mass (kg), mean (SD)			16.48 (6.40)		15.84 (4.67)		.44	
Lean body mass (kg), mean (SD)			47.97 (9.65)		49.76 (10.35)		.25	
Grip strength (kg), mean (SD)			28.61 (9.74)		31.35 (9.88)		.04^b^	
Average NRS^d^, mean (SD)			2.94 (1.55)		2.95 (1.67)		.94	
Total metabolic equivalent of task, mean (SD)			2037.45 (2097.73)		2106.87 (1954.06)		.80	
MNA^e^ total score, mean (SD)			24.41 (3.11)		24.72 (3.53)		.49	
**EORTC QLQ-C30^f^** **, mean (SD)**								
	Physical functioning	87.90 (14.94)		87.63 (20.03)		.91		
	Role functioning	88.25 (22.73)		88.76 (23.73)		.87		
	Cognitive functioning	87.19 (13.85)		88.15 (12.63)		.58		
	Emotional functioning	80.20 (20.22)		78.61 (20.30)		.56		
	Social functioning	91.23 (19.82)		92.37 (19.70)		.67		
	Fatigue	24.21 (21.92)		22.49 (21.46)		.55		
	Nausea/vomiting	7.13 (14.17)		7.03 (15.86)		.96		
	Pain	15.41 (23.16)		13.05 (23.43)		.45		
	Dyspnea	7.90 (16.72)		10.04 (19.98)		.40		
	Insomnia	20.81 (27.68)		20.88 (27.89)		.98		
	Appetite loss	12.72 (23.95)		11.65 (26.25)		.75		
	Diarrhea	13.68 (23.55)		10.04 (17.83)		.17		
	Financial difficulties	7.51 (20.05)		5.22 (16.03)		.33		
	Global health status	67.20 (23.28)		64.36 (21.04)		.33		

^a^mHealth: mobile health.

^b^*P* values <.05 were considered statistically significant.

^c^Hb: hemoglobin.

^d^NRS: Numeric Rating Scale.

^e^MNA: Mini Nutritional Assessment.

^f^EORTC QLQ-C30: European Organisation for Research and Treatment of Cancer Quality of Life Questionnaire-Core 30.

### Primary Outcome

**Figure 3:**
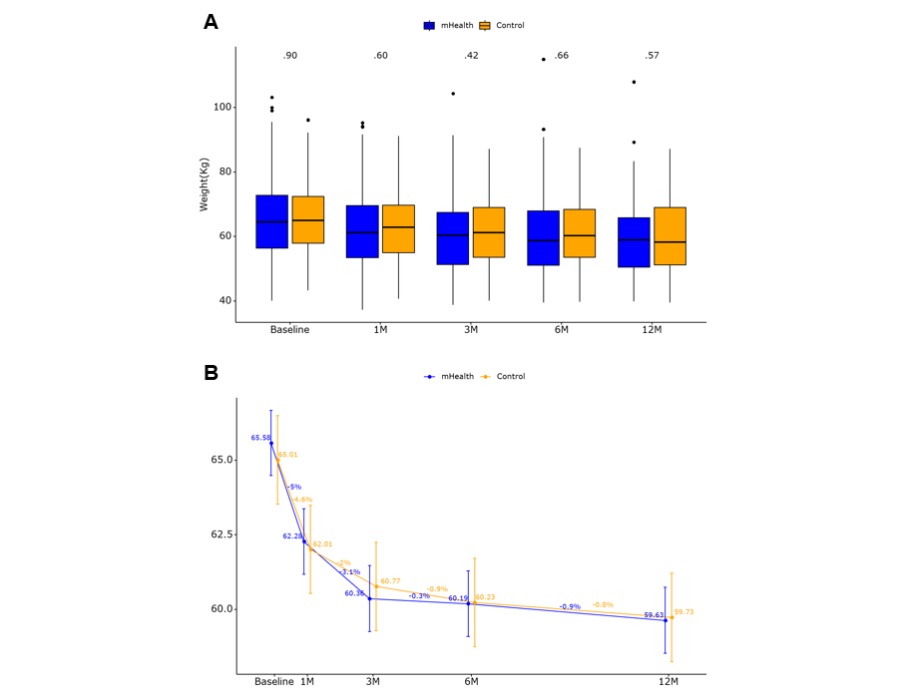
Analysis of the primary outcome with between-group comparisons at each time point. (A) Box plot showing the change in weight from baseline to 12 months postoperatively. The box represents the median (center line) and the first and third quartiles, with whiskers representing the minimum and maximum values. (B) Weight changes over the study period. Error bars represent standard errors estimated from the linear mixed model. The reported *P* value corresponds to the group × time interaction effect. Statistical significance was set at *P*<.05. mHealth: mobile health.

No significant difference was observed in body weight change between the groups over 12 months as assessed using a linear mixed model that accounted for group, time, and their interactions (Figure 3). This result remained consistent after adjusting for age, drinking, and adjuvant chemotherapy in multivariate regression (Table 2).

**Table 2:** Analysis of primary outcome.

Time point	Control	mHealth^a^	*P* value
Baseline, mean (SD)	65.54 (10.60)	65.35 (11.96)	.90
1 month, mean (SD)	62.85 (10.63)	62.05 (11.53)	.60
3 months, mean (SD)	61.46 (10.84)	60.14 (11.83)	.42
6 months, mean (SD)	60.89 (10.97)	60.16 (12.15)	.66
12 months, mean (SD)	60.20 (11.63)	59.18 (11.85)	.57
Within-group adjusted mean change from baseline to 12 months^b^, mean (95% CI)	−5.29 (−6.22 to −4.35)	−5.95 (−6.62 to −5.28)	.26^c^

^a^mHealth: mobile health.

^b^Estimated using linear mixed-effects models adjusted for age, drinking status, and adjuvant chemotherapy. Values represent the adjusted mean change in body weight from baseline to 12 months within each group.

^c^Between-group adjusted difference in mean change (mHealth-control): 0.10 (95% CI −3.01 to 3.21). A positive value reflects a greater weight loss in the mHealth group compared to the control group. Statistical significance was set at *P*<.05.

### Secondary Outcomes

The secondary outcomes of the study are summarized in Table 3. Variables analyzed included physical fitness (grip strength test, 30-second chair stand test, and 2-minute walk test), nutrition (screening and total MNA), quality of life (EORTC QLQ-C30 and EORTC QLQ-STO22), physical activity (IPAQ-SF), physical status (skeletal muscle mass, lean body mass, fat mass, BMI, hemoglobin, vitamin B_12_, and albumin), and pain intensity (average NRS). No significant group-by-time interactions or between-group differences were observed for any of these outcomes, except for the nausea/vomiting subscale of the EORTC QLQ-C30. Although the Wilcoxon rank-sum test indicated a statistically significant difference between groups (*P*<.05), both groups had identical medians and IQRs, suggesting that this result likely reflects subtle rank-based variation rather than a clinically meaningful difference in symptom burden. To further evaluate the robustness of the secondary outcome results and to complement the change-score analyses, additional longitudinal analyses were performed using GEE. The GEE results were consistent with the primary analyses, demonstrating no significant group-by-time interaction effects across all secondary outcomes (Table S5 in Multimedia Appendix 2).

**Table 3:** Analysis of secondary outcomes. For variables following a normal distribution, linear mixed-effects models were used to estimate adjusted mean changes from baseline to 12 months within each group, accounting for relevant covariates. For nonnormally distributed variables, the Wilcoxon rank-sum test was applied. “mHealth-control” indicates the estimated between-group difference in adjusted change from baseline to 12 months. A positive value reflects greater loss in the mobile health (mHealth) group compared to the control group. Statistical significance was defined as *P*<.05.

Outcome			Control		mHealth		*P* value		mHealth-control
Skeletal muscle mass, mean (95% CI)			−1.2 (−1.57 to −0.82)		−1.35 (−1.62 to −1.08)		.50		0.19 (−0.25 to 0.62)
BMI, mean (95% CI)			−2.14 (−2.50 to −1.77)		−2.13 (−2.40 to −1.87)		.99		0 (−0.43 to 0.43)
Skeletal muscle index, mean (95% CI)			−0.08 (−0.18 to 0.02)		−0.12 (−0.19 to −0.05)		.57		0.05 (−0.08 to 0.18)
Fat mass, mean (95% CI)			−3.05 (−3.91 to −2.18)		−3.90 (−4.52 to −3.28)		.12		0.74 (−0.19 to 1.67)
Lean body mass, mean (95% CI)			−1.45 (−2.26 to −0.63)		−1.66 (−2.25 to −1.08)		.67		0.33 (−0.59 to 1.25)
Grip strength, mean (95% CI)			1.48 (−0.37 to 3.33)		2.72 (1.43 to 4.00)		.28		−1.08 (−2.94 to 0.79)
Relative grip strength, mean (95% CI)			7.36 (4.36 to 10.35)		8.43 (6.34 to 10.51)		.57		−0.63 (−3.67 to 2.41)
30-second chair stand test, mean (95% CI)			5.75 (4.76 to 6.75)		5.44 (4.71 to 6.17)		.62		0.41 (−0.66 to 1.49)
2-minute walk test, mean (95% CI)			28.25 (22.22 to 34.28)		28.16 (23.80 to 32.53)		.98		1.40 (−4.51 to 7.30)
Average NRS^a^, median (IQR)			−3.00 (−4.00 to −2.00)		−3.00 (−3.00 to −2.00)		.90		0 (0 to 1)
**IPAQ-SF^b^** **, median (IQR)**									
	Total MET^c^	372.50 (−702.75 to 2204.25)		561.00 (−371.25 to 1977.00)		.87		54 (−606 to 666)	
	Vigorous MET	0.00 (0.00 to 0.00)		0.00 (0.00 to 0.00)		.25		0 (0 to 0)	
	Moderate MET	0.00 (−240.00 to 280.00)		0.00 (0.00 to 600.00)		.19		0 (0 to 240)	
	Walking MET	0.00 (−618.75 to 1178.10)		346.50 (−214.50 to 1155.00)		.59		99 (−363 to 544.5)	
**MNA^d^** **, median (IQR)**									
	MNA screening score	0.00 (−2.00 to 1.25)		0.00 (−2.00 to 1.00)		.98		0 (−1 to 1)	
	MNA total score	0.00 (−2.62 to 2.50)		0.50 (−1.50 to 2.00)		.37		0.5 (−0.5 to 1.5)	
**EORTC QLQ-C30^e^** **, median (IQR)**									
	Physical functioning	0.00 (−6.67 to 6.67)		0.00 (−6.67 to 6.67)		.88		0 (0 to 0)	
	Role functioning	0.00 (−16.67 to 0.00)		0.00 (0.00 to 0.00)		.18		0 (0 to 0)	
	Cognitive functioning	0.00 (−16.67 to 0.00)		0.00 (0.00 to 16.67)		.08		0 (0 to 0)	
	Emotional functioning	8.33 (0.00 to 16.67)		0.00 (0.00 to 16.67)		.55		0 (−8.33 to 0)	
	Social functioning	0.00 (0.00 to 0.00)		0.00 (0.00 to 0.00)		.20		0 (0 to 0)	
	Fatigue	0.00 (−11.11 to 11.11)		0.00 (−11.11 to 11.11)		.33		0 (−11.11 to 0)	
	Nausea/vomiting	0.00 (0.00 to 0.00)		0.00 (0.00 to 0.00)		.03		0 (0 to 0)	
	Pain	0.00 (−16.67 to 0.00)		0.00 (−16.67 to 0.00)		.29		0 (0 to 0)	
	Dyspnea	0.00 (0.00 to 0.00)		0.00 (0.00 to 0.00)		.75		0 (0 to 0)	
	Insomnia	0.00 (−33.33 to 0.00)		0.00 (−33.33 to 0.00)		.70		0 (0 to 0)	
	Appetite loss	0.00 (0.00 to 8.33)		0.00 (0.00 to 0.00)		.08		0 (0 to 0)	
	Constipation	0.00 (0.00 to 0.00)		0.00 (−33.33 to 0.00)		.34		0 (0 to 0)	
	Diarrhea	0.00 (0.00 to 33.33)		0.00 (0.00 to 33.33)		.52		0 (0 to 0)	
	Financial difficulties	0.00 (0.00 to 0.00)		0.00 (0.00 to 0.00)		.96		0 (0 to 0)	
	Global health status (QoL^f^)	0.00 (−10.42 to 16.67)		0.00 (−8.33 to 16.67)		.75		0 (−8.33 to 8.33)	
**EORTC QLQ-STO22^g^** **, median (IQR)**									
	Dysphagia	0.00 (−11.11 to 0.00)		0.00 (−11.11 to 0.00)		.43		0 (0 to 0)	
	Pain	0.00 (−16.67 to 8.33)		−8.33 (−16.67 to 8.33)		.58		0 (−8.33 to 0)	
	Reflux symptoms	0.00 (−11.11 to 11.11)		0.00 (−11.11 to 0.00)		.49		0 (0 to 0)	
	Eating restrictions	0.00 (−16.67 to 8.33)		0.00 (−14.58 to 8.33)		.85		0 (−8.33 to 8.33)	
	Anxiety	0.00 (−11.11 to 11.11)		0.00 (−11.11 to 11.11)		.48		0 (−11.11 to 0)	
	Dry mouth	0.00 (−33.33 to 0.00)		0.00 (0.00 to 0.00)		.56		0 (0 to 0)	
	Taste	0.00 (−8.33 to 0.00)		0.00 (−33.33 to 0.00)		.77		0 (0 to 0)	
	Body image	0.00 (0.00 to 0.00)		0.00 (0.00 to 0.00)		.58		0 (0 to 0)	
	Hair loss	16.67 (0.00 to 33.33)		0.00 (0.00 to 25.00)		.61		0 (−66.67 to 33.33)	
Hb^h^, mean (95% CI)			−0.80 (−1.70 to 0.10)		−0.60 (−1.40 to 0.30)		.56		0.1 (−0.3 to 0.6)
Vitamin B_12_, mean (95% CI)			−215.00 (−362.00 to −27.00)		−210.50 (−325.75 to 63.50)		.56		60.5 (−221 to 410)
Albumin, mean (95% CI)			−0.10 (−0.20 to 0.10)		0.00 (−0.30 to 0.17)		.42		0 (−0.1 to 0.2)

^a^NRS: Numeric Rating Scale.

^b^IPAQ-SF: International Physical Activity Questionnaire-Short Form.

^c^MET: metabolic equivalent of task.

^d^MNA: Mini Nutritional Assessment.

^e^EORTC QLQ-C30: European Organisation for Research and Treatment of Cancer Quality of Life Questionnaire-Core 30.

^f^QoL: quality of life.

^g^EORTC QLQ-STO22: European Organisation for Research and Treatment of Cancer Quality of Life Questionnaire-Stomach 22.

^h^Hb: hemoglobin.

### eHEALS

eHealth literacy was assessed only at baseline in the intervention group using the eHEALS. The mean score was 29.8 (SD 4.05) out of 40, indicating a moderate level of eHealth literacy. This suggests that while participants were likely capable of independently searching for health information online, they may have had limitations in evaluating accuracy and reliability.

### Compliance

**Figure 4:**
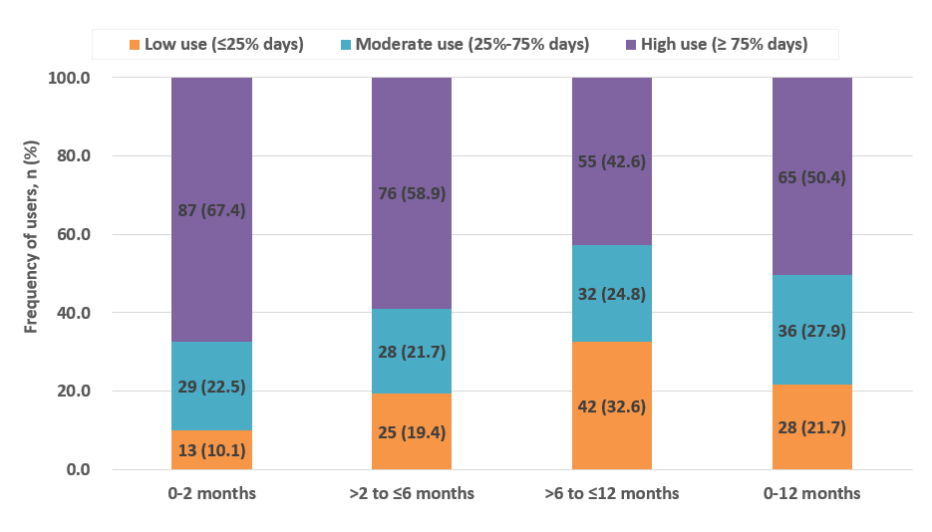
Compliance with wearable device–linked mobile app use over 12 months. Stacked bars show the number and proportion of participants with low (≤25%), moderate (25%-74%), and high (≥75%) usage across 4 time intervals: 0-2 months, 2-6 months, 6-12 months, and the overall 12-month period.

Compliance with wearable device–linked mobile app use over 12 months is presented in Figure 4. Usage was categorized as high (≥75% of days), moderate (25%-74% of days), and low (≤25% of days). Compliance was highest during the initial 0-2 months, followed by a gradual decline during the 2- to 6-month and 6- to 12-month intervals. Nevertheless, overall adherence was favorable, with approximately 78.3% (101/129) of the participants demonstrating moderate to high use throughout the study period.

### Association Between Adherence and Weight Change

**Figure 5:**
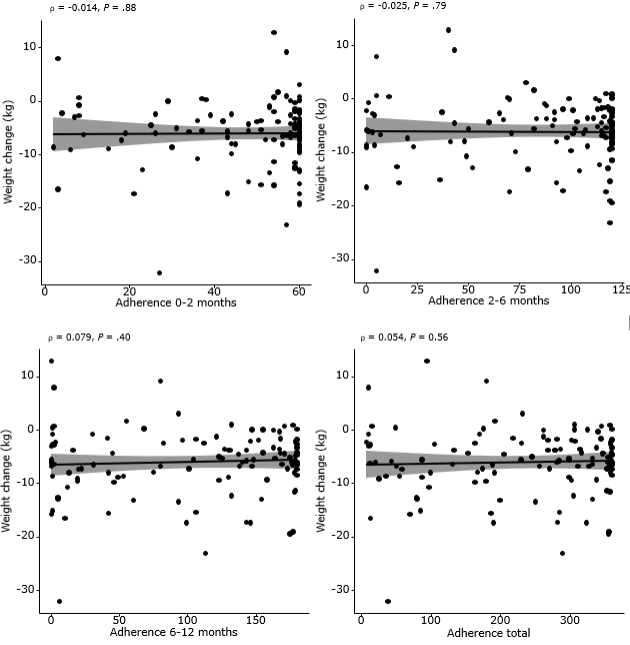
Correlation between intervention adherence and 12-month weight change from baseline.

To explore a potential dose-response relationship, we analyzed the correlation between app adherence and 12-month weight change. Adherence was calculated separately for 3 time intervals (0-2 months, 2-6 months, and 6-12 months) and for the total intervention period. As shown in Figure 5, Spearman correlation analyses revealed no statistically significant associations between adherence and weight change in any period: ρ=–0.014 (*P*=.88) for 0-2 months; ρ=–0.025 (*P*=.79) for 2-6 months; ρ=0.079 (*P*=.40) for 6-12 months; ρ=0.054 (*P=*.56) for the total study period. These results suggest that higher app engagement levels were not significantly associated with attenuated postoperative weight loss in this cohort.

### App-Based Adherence Measures

Among the 131 participants in the intervention group who completed the 12-month follow-up, data from 129 were included in the adherence analysis. Individuals who discontinued app use or withheld consent for data access at the time of data retrieval were excluded. App usage patterns over the 12-month intervention period are summarized in Table 4. Participants recorded exercise on an average of 18.2 (SD 10.0) days per month, with a mean duration of 56.9 (SD 26.9) minutes per exercise day. The number of step count log days remained relatively stable throughout the follow-up (overall mean 24.2, SD 8.7 days per month), with an average of 8491.1 (SD 3765.5) steps per day. Weight and diet logs were entered on an average of 9.9 (SD 10.9) and 22.7 (SD 8.8) days per month, respectively. App access was recorded on 21.3 (SD 10.0) days per month. While the frequency of exercise logging was higher in the earlier months, average exercise duration per exercise day and daily step counts showed an increasing trend over time, suggesting improved engagement in physical activity among users who continued to log their data.

**Table 4:** Monthly digital intervention engagement: exercise, step count, weight logs, diet logs, and app use (n=129). Exercise logs include any session (free-form, video-guided, or manually entered) lasting at least 5 minutes. Aerobic exercise refers to sessions lasting ≥10 minutes. Step counts, weight, and dietary logs were self-reported via the digital platform.

Month	Exercise log (days), mean (SD)	Exercise duration per log day (min), mean (SD)	Step count logged (days), mean (SD)	Step count per log day (steps), mean (SD)	Weight log (days), mean (SD)	Diet log (days), mean (SD)	App access (days), mean (SD)
1	14.3 (10.7)	42.4 (28.6)	22.7 (9.2)	7435.4 (3751.4)	8.4 (8.9)	22.0 (9.7)	22.4 (9.6)
2	19.8 (10.0)	44.4 (26.2)	24.6 (8.8)	8428.4 (4138.4)	10.7 (10.8)	24.4 (7.4)	22.6 (9.3)
3	19.2 (10.4)	46.4 (23.2)	23.8 (9.0)	8099.8 (3592.5)	10.7 (11.6)	23.1 (9.3)	22.3 (9.4)
4	18.7 (10.1)	58.6 (27.3)	23.9 (9.1)	8327.0 (3535.0)	10.7 (11.4)	23.5 (8.9)	21.4 (10.2)
5	19.6 (9.3)	63.1 (28.2)	24.4 (8.7)	8836.8 (4294.3)	10.3 (11.4)	23.6 (8.1)	22.0 (9.7)
6	19.7 (9.1)	61.2 (25.2)	24.5 (8.3)	8314.8 (3480.2)	10.3 (11.1)	22.9 (8.4)	21.3 (10.0)
7	18.6 (9.5)	62.2 (24.5)	23.7 (8.7)	8310.9 (3599.6)	10.0 (11.3)	22.4 (8.8)	20.8 (10.4)
8	17.9 (9.7)	64.3 (23.3)	25.1 (7.8)	8947.9 (3803.8)	9.3 (10.8)	22.0 (9.1)	20.7 (10.1)
9	18.4 (9.5)	65.5 (25.5)	25.0 (8.4)	9316.1 (3634.5)	9.5 (11.2)	22.1 (8.8)	21.0 (9.6)
10	18.6 (9.2)	64.8 (23.1)	25.3 (8.5)	9261.3 (3552.2)	10.0 (11.1)	21.8 (8.8)	20.6 (10.4)
11	17.0 (10.1)	64.8 (26.5)	25.2 (7.9)	9071.0 (3547.0)	9.8 (11.1)	21.7 (8.9)	19.8 (10.6)
12	17.5 (10.1)	62.0 (21.2)	23.5 (9.3)	8550.0 (3694.4)	9.5 (11.0)	21.1 (9.3)	19.2 (10.9)
Overall	18.2 (10.0)	56.9 (26.9)	24.2 (8.7)	8491.1 (3765.5)	9.9 (10.9)	22.7 (8.8)	21.3 (10.0)

### Satisfaction Survey

A customized satisfaction survey was conducted with participants in the intervention group at the 6-month follow-up (n=113). Overall, responses reflected a high degree of satisfaction with the digital health care service (Table 5).

Among the assessed domains, perceived effectiveness received the most favorable ratings, with average scores ranging from 4.0 (SD 0.6) to 4.1 SD (0.7). These results suggest that participants felt the service contributed meaningfully to their efforts to monitor and manage their health. Items addressing usability also performed well (means ranging from 3.9, SD 0.8 to 4.0, SD 0.9), indicating that participants generally found the app intuitive and easy to use.

In the domain of content adequacy, scores were slightly lower, with the item evaluating whether the service included all essential features for health management receiving a mean score of 3.7 (SD 0.8). Many participants noted that the exercise and dietary management tools were especially useful. On the other hand, several respondents identified dietary tracking as an area requiring further refinement.

**Table 5:** Satisfaction survey results at 6-month follow-up (intervention group; n=113).

Survey question			Mean (SD)
**Use and access**			
	The service provided sufficient in-app instructions on how to use its features.	4.0 (0.9)	
	Understanding how to operate the service was straightforward^a^.	3.9 (0.8)	
	I was able to easily locate and use the functions I needed.	3.9 (0.8)	
**Content adequacy**			
	The service included all the essential features required for managing my health.	3.7 (0.8)	
	The service offered sufficient and helpful information that supported my health management and lifestyle improvement^a^.	3.9 (0.7)	
**Satisfaction**			
	Overall, how satisfied are you with the healthcare service you used?^a^	3.9 (0.7)	
	Do you intend to continue using the healthcare service in the future?^a^	3.8 (0.9)	
	Would you recommend this healthcare service to other patients?^a^	3.9 (0.8)	
**Perceived effectiveness**			
	Since using the service, have you monitored your health status more regularly?	4.0 (0.6)	
	Has your interest in managing your personal health increased after using the service?	4.0 (0.6)	
	Was the service useful in supporting your personal health management?	4.0 (0.6)	
	Do you consider the service a suitable method for managing your condition?	4.1 (0.7)	

^a^Included a follow-up question to elaborate on their answers.

## Discussion

### Principal Findings

This study investigated the long-term effects of a personalized, stage-adjusted digital health program combining exercise and nutritional interventions in patients with GC, delivered for 12 months following surgery. While overall satisfaction with and compliance with the digital health intervention were relatively high, the intervention group did not show a statistically significant benefit in mitigating postoperative body weight loss compared to the control group. Secondary outcomes, including body composition, BMI, physical fitness level, nutrition, and quality of life, did not demonstrate meaningful improvements across time points. The overall consistency between the results of nonparametric tests and GEE analyses suggests that the absence of statistically significant group-by-time interaction effects is unlikely due to model selection. This concordance reinforces the robustness and validity of our findings related to the secondary outcomes.

The hypothesized mechanisms by which a digital health program combining exercise and nutrition might influence postoperative weight loss after gastrectomy can be conceptualized in 3 interrelated domains: behavioral activation, metabolic/nutritional modulation, and muscle-preserving functional support. The app and wearable device supported self-monitoring, goal setting, and feedback, promoting adherence to physical activity and dietary goals. These behavioral components are especially valuable during early recovery, when fatigue and reduced intake are common. In addition, prior studies in populations with cancer support the effectiveness of such digital tools in sustaining health behaviors [43]. Postoperative weight loss in patients with GC is a multifactorial process influenced by mechanical, hormonal, and metabolic changes (eg, increased catabolism and inflammatory responses). These alterations result in decreased oral intake, impaired nutrient absorption, and significant losses in skeletal muscle mass and body weight. Structured digital interventions can modulate these adverse trajectories by optimizing caloric intake, preserving lean body mass, and minimizing nutrient deficiencies [44,45]. Furthermore, exercise prescriptions were tailored using cancer guidelines, combining aerobic and resistance training to mitigate sarcopenia and preserve function [24]. The individualized prescriptions targeted aerobic and resistance training intensity using patient-specific maximum heart rate and stage-based progression, aiming to preserve lean mass and physical function. Digital delivery enabled sustained support during the high-risk postdischarge period, allowing flexible, remote adjustments to care without increasing burden [10].

Nevertheless, several factors may explain why the 12-month digital health intervention did not significantly alter the postoperative weight loss trajectory in patients with GC compared to conventional education-based rehabilitation.

### Reach

Our trial enrolled 257 patients after curative resection for stage I-III GC. As noted in the manuscript, the study population had relatively preserved nutritional status (mean BMI 24.05, SD 3.36 kg/m^2^ in the intervention group, 24.05, SD 2.79 kg/m^2^ in the control group; *P*=.99), with only 2 participants (BMI 16.1 kg/m^2^ and 16.2 kg/m^2^) having BMI <17 kg/m^2^. This indicates that the study population predominantly comprised individuals at a relatively lower baseline risk of nutritional compromise, potentially omitting those at the highest nutritional risk who may have derived the greatest benefit from the intervention. In addition, although current clinical guidelines emphasize dietary practices to prevent complications [46,47], many patients fail to adopt them due to insufficient cognitive factors, such as limited risk perception, weak motivation, or limited expectations of benefit [48-50]. A study in patients with upper gastrointestinal cancer showed that weight loss was attenuated only with telephone counseling, while mobile app–based support alone was insufficient to ensure nutritional adequacy [51]. Moreover, a Korean survey reported that the general public perceived digital therapeutics less favorably than health care professionals [52].

### Effectiveness

Although the personalized mHealth program was safe and well-received—with high early-phase adherence and no reported adverse events—it did not yield statistically significant differences in weight change or secondary clinical outcomes (eg, quality of life, physical function, and nutritional biomarkers). This highlights the importance of distinguishing between biological efficacy and intervention usability/satisfaction, especially in patient populations with a low baseline risk. GC has a unique biological trajectory compared with that of other malignancies. Patients undergoing gastrectomy typically exhibit substantial changes in body composition, characterized by marked reductions in body weight during the first 6 months, followed by gradual stabilization [10,53]. One study reported that the most pronounced weight changes occurred within 6-12 months, with stabilization or recovery extending up to 2 years after surgery [1]. This biphasic course may be resistant to reversal even with structured digital interventions. Postoperative weight loss in GC is multifactorial, driven by inflammatory responses, reduced intake, and decreased physical activity. Tumor-related malnutrition and immune dysfunction are further aggravated by radical surgery, underscoring the need for both nutritional and immune-supportive interventions [54,55]. This is consistent with findings that most oral nutritional supplementation trials in GC have not shown consistent benefits in preventing postoperative weight loss [13,43,44]. While GC and colorectal cancer share gastrointestinal origins, GC is associated with poorer survival and more aggressive progression due to differences in the underlying metabolic pathways [56-58]. Although recent randomized controlled trials in colorectal cancer showed quality-of-life improvements through mHealth, these results may not be generalizable to GC due to these fundamental biological differences.

In addition, the conservative nature of the intervention, largely driven by safety considerations, may have limited its efficacy. Unlike supervised rehabilitation programs, the current unsupervised digital program required cautious exercise prescriptions. Moreover, both groups received comprehensive standard care, narrowing the margin for additional benefit.

Another potential contributing factor is the lack of real-time symptom management and psychological support, as the program focused solely on exercise and nutrition. Psychological support is known to be critical in postoperative quality of life for patients with GC, breast cancer, and esophagogastric cancer [59-63]. However, due to the COVID-19 pandemic, it was not feasible to incorporate family or peer-based support. Future mHealth programs should incorporate humanized psychological support and caregiver-focused components [3,60,62].

### Adoption and Implementation

The intervention was implemented across multiple hospitals and integrated into routine postgastrectomy care, delivered through a mobile app and wearable device. The program was initiated within 1 week after surgery and maintained for 12 months, aligning with critical recovery periods. However, we observed a progressive decline in engagement, particularly during the 6- to 12-month period—a common challenge in long-term digital interventions. While this low-touch, patient-led design ensured feasibility and safety, the absence of active supervision may have limited behavior change, particularly in patients with lower health literacy or motivation. Although exercise and nutrition prescriptions were algorithmically tailored, adherence to these prescriptions in real-world settings was not directly assessed, limiting our understanding of implementation fidelity.

### Maintenance

Although the intervention spanned 12 months, true maintenance—sustained behavior changes and functional improvement—often extends beyond this time frame. Systematic reviews of web-based digital health interventions have demonstrated efficacy attenuation over extended time frames due to digital fatigue, with short-term benefits failing to translate into sustained long-term outcomes [64-67]. Therefore, further research in GC is needed to investigate strategies that can maintain high patient engagement over periods exceeding 2 years, as well as including approaches such as gamification to enhance motivation [68].

To maximize the effectiveness of digital health, it is essential to deliver the right content to the right patients at the right time. For example, an Internet of Things–based lifestyle intervention in prostate cancer demonstrated benefits among patients receiving androgen deprivation therapy or those with a high symptom burden, whereas effects were inconsistent in broader populations and across different intervention durations [69,70]. In our study, the digital health program, initiated immediately after gastrectomy and maintained for 12 months at a safe intensity, might have served mainly as supportive monitoring without conferring superior effects on the biological course of GC. Therefore, further research on precise targeting is warranted to tailor intervention content to the specific characteristics of each cancer type.

### Strengths and Limitations

This study has several notable strengths. First, to the best of our knowledge, this is the largest and longest randomized controlled trial to date to evaluate a mHealth and nutrition platform in patients with GC, enrolling 257 participants with a follow-up period of 12 months. Second, the digital health program was highly personalized and dynamically adjusted based on the patient’s treatment phase and clinical condition. Third, the program was initiated within 1 week after surgery, enabling early rehabilitation and nutritional support during a critical recovery window. Fourth, the program was safe and well-received, with no adverse events and high levels of satisfaction and adherence. This reinforces its clinical relevance as a feasible and acceptable model of digital rehabilitation. Fifth, although mHealth interventions have been reported to be broadly beneficial in cancer rehabilitation, this study allowed for a focused examination of their applicability to GC. Our findings suggest a novel viewpoint that a digital health program centered solely on nutrition and exercise for 12 months may have limited effectiveness in the postoperative GC population, unlike some other cancers. Finally, our study adhered to CONSORT/SPIRIT standards.

This study has several limitations. First, this was an open-label design that was not free of bias. However, this reflects real-world clinical settings, thereby enhancing the external validity of the findings. In addition, compliance gradually declined at 2- to 6-and 6- to 12-month intervals. Nevertheless, overall adherence remained favorable. Weight change after gastrectomy for GC is most pronounced within the first 6-12 months, with subsequent stabilization or improvement observed up to 2 years postoperatively [1,71]. Given that our study was limited to a 12-month follow-up period, the long-term recovery trajectory could not be fully characterized, considering the physiology of GC. Moreover, most participants entered the study with relatively preserved nutritional status, which may have contributed to a ceiling effect and reduced the magnitude of observable benefit, particularly for nutritional outcomes. This profile likely reflects a selection bias that patients with greater frailty or poor performance status may be less likely to enroll or remain eligible. Furthermore, as the study was powered based solely on the primary outcome with an assumed moderate effect size, it may have been underpowered to detect smaller but clinically meaningful effects in secondary end points. Given the large number of secondary outcomes assessed, the absence of multiplicity adjustment may increase the risk of type I error (false positives). At the same time, because the sample size was not specifically calculated to detect effects in secondary variables, the study may also be subject to type II error (false negatives) for outcomes with small effect sizes. Accordingly, analyses of secondary outcomes were exploratory in nature, and no adjustments were made for multiple comparisons. Therefore, findings related to secondary end points should be interpreted with caution and regarded as hypothesis-generating rather than confirmatory. While adherence metrics were reported, key implementation outcomes related to intervention reach (eg, recruitment yield, refusal reasons, and population representativeness) were not quantitatively captured. This represents a limitation in fully characterizing the implementation context and may constrain the interpretation of broader applicability. Finally, participants were recruited from a single monoracial country. Future studies should aim to recruit more diverse populations to enhance global generalizability.

### Conclusion

In summary, this randomized controlled trial showed that a personalized digital health program integrating exercise and nutrition is safe, feasible, and associated with high satisfaction and adherence among patients with GC in the early postoperative period. However, the intervention did not produce measurable benefits over standard education in postoperative weight change or other functional and nutritional outcomes. These results suggest that when applied broadly across all postoperative patients, digital health tools may function primarily as supportive monitoring platforms rather than interventions that biologically augment recovery. Future research is needed to identify subgroups that may benefit most, optimize intervention timing and intensity, and determine how digital programs can complement existing rehabilitation pathways.

## Appendix

Multimedia Appendix 1 Additional tables.

Multimedia Appendix 2 CONSORT-EHEALTH checklist.

## Abbreviations

CONSORT: Consolidated Standards of Reporting Trials
eHEALS: eHealth Literacy Scale
EORTC QLQ-C30: European Organisation for Research and Treatment of Cancer Quality of Life Questionnaire-Core 30
EORTC QLQ-STO22: European Organisation for Research and Treatment of Cancer Quality of Life Questionnaire-Stomach 22
GC: gastric cancer
GEE: generalized estimating equation
IPAQ-SF: International Physical Activity Questionnaire-Short Form
MET: metabolic equivalent of task
mHealth: mobile health
MNA: Mini Nutritional Assessment
NRS: Numeric Rating Scale
SPIRIT: Standard Protocol Items: Recommendations for Interventional Trials
THR: target heart rate

## Data Availability

The datasets used and/or analyzed in this study are available from the corresponding author upon reasonable request.
