# Supplementary material for: Effectiveness of a Personalized Digital Exercise and Nutrition Rehabilitation Program in Postoperative Patients With Gastric Cancer: Randomized Controlled Trial
Source: JMIR Mhealth Uhealth. 2026 Feb 26;14:e85795. doi: 10.2196/85795 (PMC12982962; doi:10.2196/85795)
Supplement: Multimedia Appendix 1 [file mhealth_v14i1e85795_app1.docx]

**Table S1.** Decision rules used in the personalized exercise stage assignment algorithm

| **Step** | **Decision Rule** | **Input** | **Action** |
| --- | --- | --- | --- |
| **Step 1** | Initial Stage Assignment | Postoperative week (based on surgery date) | Assign base exercise stage according to a predefined progression table |
| **Step 2** | Chemotherapy Adjustment | Patient reports initiation of chemotherapy | Automatically downgrade to a lower-intensity stage (stretching-focused), regardless of postoperative week |
| **Step 3** | Monitoring Window | Exercise data from the previous 14 days | Evaluate adherence and perceived exertion to determine need for adjustment |
| **Step 4** | Downward Adjustment Criteria | RPE* ≥ 'very hard', or 'hard' (for consecutive days) | Downgrade exercise stage before any other adjustments |
| **Step 5** | Upward Adjustment Criteria | RPE* = 'light' or 'moderate' (for consecutive days) | Upgrade stage, limited by the maximum allowable stage for current postoperative week |
| **Step 6** | Behavioral Feedback Loop | Recent exercise duration and frequency | Use adherence data to confirm or modify stage for subsequent sessions |

***** RPE: Rating of Perceived Exertion

**Table S2.** Decision rules used in the personalized nutrition prescription algorithm

| **Step** | **Decision Rule** | **Input** | **Action** |
| --- | --- | --- | --- |
| **Step 1** | Determine Operation Type | Surgical procedure (Total vs Subtotal gastrectomy) | Categorize patient into total or subtotal gastrectomy group |
| **Step 2** | Identify Postoperative Phase | Weeks since surgery | Assign to Recovery (1–4w), Adaptation (5–12w), or Maintenance (≥13w) phase |
| **Step 3** | Estimate Activity Index & Caloric Needs | Age (<65 vs. ≥65 years) and standard body weight (kg) | Calculate recommended daily caloric intake (kcal/day) based on age and standard body weight |
| **Step 4** | Define Nutrient Composition | Standard body weight, total kcal needs, comorbidities | Prescribe: Protein = 1.2g/kg, Fat = 20% of kcal/day, and tailor micronutrients based on comorbid conditions |

**Table S3.** Functions of the mobile app and key characteristics

| **Functions** | **Key characteristics** |
| --- | --- |
| Expert consultation | - The program enables users to consult exercise and nutrition specialists through multimedia formats, including text messages, voice recordings, and images. Consultations are limited to lifestyle management and do not extend to clinical symptoms. Responses are typically provided within 24 h. - The expert team includes licensed dietitians and certified exercise physiologists. |
| Second doctor Journal | - This feature delivers educational content focused on exercise, nutrition, and disease management. Information is based on the surgical procedure, treatment modality, and comorbidities. |
| Exercise management | - An individualized aerobic exercise regimen is provided, adjusted for each user’s treatment status, including chemotherapy. - A progressive weekly plan combines video-guided stretching and resistance exercises. Intensity levels are personalized based on self-reported ratings of perceived exertion post-exercise. |
| Diet management | - Users can log their daily food intake via speech recognition or text. The system provides immediate feedback on caloric intake, macronutrient balance (especially protein and fat), and food diversity, categorized as insufficient, appropriate, or excessive. - Initial caloric recommendations are derived from baseline weight and are subsequently adapted based on gastrectomy type (total or subtotal), age, and body mass index (BMI). - From week 13 onward, dietary suggestions are further refined according to comorbid conditions and portion recommendations for each eating occasion. |
| Self-symptom tracking | - Weekly tracking of postprandial symptoms, including features indicative of dumping syndrome, is facilitated through a scoring system. This allows users to observe trends over time. |
| Physical activity management | - The platform sets individualized step count targets (e.g., 5,000 steps/day) and monitors heart rate and daily energy expenditure. Visual feedback is provided on step counts, categorized by activity type, as well as corresponding calorie consumption and heart rate trends. |
| Comorbidity and Weight management | - Users receive alerts regarding their weight status relative to standard ranges. - The system also displays target values for blood pressure and glucose, informed by current clinical guidelines for comorbidity management. |

**Table S4.** Summary of the study visit schedule: baseline screening, assessment, and follow-up during study visits

|  | | **STUDY PERIOD** | | | | |
| --- | --- | --- | --- | --- | --- | --- |
|  |  | **Post-allocation** | | | | |
| **TIMEPOINT** | | **Post op**  **(+1w)** | **1-month**  **(±2w)** | **3-month**  **(±1m)** | **6-month**  **(±2m)** | **12-month**  **(±2m)** |
|  | **ENROLLMENT:** |  |  |  |  |  |
| **1** | **Eligibility screen** | X |  |  |  |  |
| **2** | **Informed consent** | X |  |  |  |  |
| **3** | **Allocation** | X |  |  |  |  |
| **4** | **Demographic characteristics** | X |  |  |  |  |
| **5** | **Medical history** | X |  |  |  |  |
| **6** | **Health lifestyle** | X |  |  |  |  |
| **7** | **eHealth Literacy Scale** ^a^ | X |  |  |  |  |
|  | **INTERVENTIONS:** |  |  |  |  |  |
|  | **Personalized digital therapeutic (intervention) group** |  |  |  |  |  |
|  | **Control group** |  |  |  |  |  |
|  | **ASSESSMENTS:** |  |  |  |  |  |
| **8** | **Complication** |  | X | X | X | X |
| **9** | **Hb, albumin, Vitamin B12** | X |  |  | X | X |
| **10** | **Height & weight** | X | X | X | X | X |
| **11** | **Body composition** | X | X | X | X | X |
| **12** | **Mini Nutritional Assessment** | X | X | X | X | X |
| **13** | **IPAQ-SF** | X | X | X | X | X |
| **14** | **EORTC QLQ-C30** | X | X | X | X | X |
| **15** | **EORTC QLQ-STO22** |  | X | X | X | X |
| **16** | **Numeric Rating Scale** | X | X | X | X | X |
| **17** | **Grip strength** | X | X | X | X | X |
| **18** | **30 s chair stand test** |  | X | X | X | X |
| **19** | **2 min walk test** |  | X | X | X | X |
| **20** | **Satisfaction questionnaire** ^a^ |  |  |  | X |  |

IPAQ-SF: International Physical Activity Questionnaire-Short Form; EORTC QLQ-C30, European Organization for Research and Treatment of Cancer Quality of Life Questionnaire-Core 30; EORTC QLQ-S22, European Organisation for Research and Treatment of Cancer Quality of Life Questionnaire-Stomach 22; Hb, Hemoglobin

^a^ Personalized digital therapeutic (intervention) group only.

**Table S5.** Results of generalized estimating equation models assessing group-by-time interaction effects for secondary outcomes with non-normal distributions

|  | **Control** | **mHealth** | ***P* value** |
| --- | --- | --- | --- |
| Average NRS | -2.60 (-3.03 ~ -2.17) | -2.70 (-2.99 ~ -2.42) | 0.703 |
| IPAQ-SF |  |  |  |
| Total MET | 569.21 (-103.35 ~ 1241.77) | 618.23 (164.24 ~ 1072.23) | 0.906 |
| Vigorous MET | 236.42 (-71.41 ~ 544.26) | 1.41 (-237.01 ~ 239.84) | 0.237 |
| Moderate MET | 50.71 (-194.70 ~ 296.13) | 156.88 (-19.36 ~ 333.13) | 0.491 |
| Walking MET | 282.07 (-136.09 ~ 700.23) | 459.94 (152.71 ~ 767.16) | 0.501 |
| MNA screening score | -0.18 (-0.83 ~ 0.47) | -0.13 (-0.59 ~ 0.32) | 0.909 |
| MNA total score | -0.07 (-1.20 ~ 1.07) | 0.45 (-0.25 ~ 1.16) | 0.446 |
| EORTC QLQ-C30 | |  |  |
| Physical functioning | 3.60 (-1.42 ~ 8.62) | 3.79 (0.93 ~ 6.65) | 0.949 |
| Role functioning | 0.19 (-6.01 ~ 6.39) | 4.56 (0.19 ~ 8.94) | 0.258 |
| Cognitive functioning | -2.43 (-6.95 ~ 2.09) | 1.18 (-2.13 ~ 4.49) | 0.206 |
| Emotional functioning | 6.38 (-0.02 ~ 12.77) | 7.16 (3.18 ~ 11.15) | 0.838 |
| Social functioning | -1.34 (-7.06 ~ 4.39) | 2.76 (-1.23 ~ 6.75) | 0.250 |
| Fatigue | 2.43 (-4.48 ~ 9.34) | -2.31 (-6.77 ~ 2.16) | 0.259 |
| Nausea/Vomiting | 1.13 (-4.33 ~ 6.59) | -1.39 (-4.50 ~ 1.73) | 0.432 |
| Pain | -5.37 (-11.52 ~ 0.79) | -9.11 (-13.39 ~ -4.84) | 0.327 |
| Dyspnea | -4.56 (-10.10 ~ 0.98) | -3.26 (-6.85 ~ 0.32) | 0.700 |
| Insomnia | -5.36 (-13.50 ~ 2.78) | -5.78 (-11.46 ~ -0.10) | 0.934 |
| Appetite loss | 1.80 (-5.86 ~ 9.47) | -5.63 (-10.35 ~ -0.91) | 0.106 |
| Constipation | -4.15 (-9.78 ~ 1.49) | -4.44 (-9.19 ~ 0.30) | 0.937 |
| Diarrhea | 7.31 (0.51 ~ 14.11) | 3.51 (-1.63 ~ 8.66) | 0.382 |
| Financial difficulties | 1.20 (-4.09 ~ 6.49) | -1.22 (-5.46 ~ 3.01) | 0.483 |
| Global health status(QoL) | 4.89 (-1.19 ~ 10.97) | 5.50 (0.78 ~ 10.22) | 0.876 |
| EORTC QLQ-STO22 |  |  |  |
| Dysphagia | -3.33 (-6.74 ~ 0.09) | -6.06 (-8.74 ~ -3.39) | 0.215 |
| Pain | -2.54 (-7.53 ~ 2.46) | -5.97 (-9.32 ~ -2.62) | 0.262 |
| Reflux symptoms | 0.81 (-3.76 ~ 5.39) | -3.29 (-6.55 ~ -0.03) | 0.152 |
| Eating restrictions | -3.52 (-8.01 ~ 0.97) | -6.39 (-9.90 ~ -2.88) | 0.323 |
| Anxiety | 2.57 (-4.10 ~ 9.24) | -1.39 (-6.21 ~ 3.43) | 0.346 |
| Dry mouth | -1.07 (-9.25 ~ 7.11) | -7.57 (-12.84 ~ -2.30) | 0.190 |
| Taste | -5.24 (-10.63 ~ 0.16) | -8.47 (-12.56 ~ -4.39) | 0.348 |
| Body image | 2.06 (-6.11 ~ 10.22) | -2.85 (-8.85 ~ 3.16) | 0.343 |
| Hair loss | 3.33 (-18.67 ~ 25.33) | 4.55 (-10.92 ~ 20.01) | 0.929 |
| Hb | -0.22 (-0.87 ~ 0.44) | -0.26 (-0.66 ~ 0.13) | 0.906 |
| Vitamin B12 | -189.42 (-329.70 ~ -49.15) | -16.44 (-379.78 ~ 346.89) | 0.382 |
| Albumin | -0.10 (-0.21 ~ 0.00) | -0.00 (-0.07 ~ 0.07) | 0.125 |
| Values are presented as mean (95% confidence interval). Estimated difference (95% CI) indicates the adjusted mean change in variables from baseline to 12 months within each group, calculated using linear mixed-effects models accounting for relevant covariates. Statistical significance was set at *P*<.05. NRS, Numeric Rating Scale; IPAQ-SF, International Physical Activity Questionnaire-Short Form; MNA, Mini Nutritional Assessment; EORTC QLQ-C30, European Organisation for Research and Treatment of Cancer Quality of Life Questionnaire-Core 30; EORTC QLQ-S22, European Organisation for Research and Treatment of Cancer Quality of Life Questionnaire-Stomach 22; Hb, hemoglobin | | | |
